# *Lycium barbarum* polysaccharide modulates gut microbiota to alleviate rheumatoid arthritis in a rat model

**DOI:** 10.1038/s41538-022-00149-z

**Published:** 2022-07-21

**Authors:** Wenjia Lai, Chunyan Wang, Renfa Lai, Xichun Peng, Jianming Luo

**Affiliations:** 1 The First Clinical Medical College of Jinan University, Jinan University, Guangzhou, 510630 Guangdong China; 2 Department of Food Science and Engineering, Jinan University, Guangzhou, 510630 Guangdong China; 3 The First Affiliated Hospital of Jinan University, Clinical Research Platform for Interdiscipline of Stomatology, Jinan University, Guangzhou, 510630 Guangdong China

**Keywords:** Microbiome, Polysaccharides

## Abstract

Rheumatoid arthritis (RA) seriously impairs the quality of life of sufferers. It has been shown that *Lycium barbarum* polysaccharide (LBP), a natural active indigestible ingredient with medicinal and edible functions, can effectively relieve RA, however, whether this effect is related to gut microbiota is not known. This study aimed to explore the RA alleviating mechanism of LBP mediated by gut microbiota using a collagen-induced arthritis rat model. The results showed that LBP significantly changed the gut microflora structure accompanied with the RA alleviation. Specifically, a LBP intervention reduced the relative abundance of *Lachnospiraceae*_NK4A136_group and uncultured_bacterium_f_*Ruminococcaceae* and significantly increased the abundance of *Romboutsia*, *Lactobacillus*, *Dubosiella* and *Faecalibaculum*. The mRNA contents of several colonic epithelial genes including *Dpep3*, *Gstm6*, *Slc27a2*, *Col11a2*, *Sycp2*, *SNORA22*, *Tnni1*, *Gpnmb*, *Mypn* and *Acsl6*, which are potentially associated to RA, were down-regulated due to the DNA hypermethylation, possibly caused by the elevating content of a bacterial metabolite S-adenosyl methionine (SAM). In conclusion, our current study suggests that LBP alleviated RA by reshaping the composition of intestinal microflora which may generate SAM, inducing DNA hypermethylation of RA-related genes in the host intestinal epithelium and subsequently reducing their expression.

## Introduction

Rheumatoid arthritis (RA) is a chronic autoimmune disease of unknown etiology, and characterized by infiltration of activated immune cells and production of inflammatory factors leading to synovial hyperplasia and pannus, as well as the destruction of cartilage and joints. Clinically, RA manifested as joint swell and stiffness, musculoskeletal pain and irreversible bone damage, and even permanent disability, which can seriously affect the quality of life^1–3^. At present, due to the potential side effects of drugs on the clinical therapy of RA, researchers are actively exploring other treatments. The development of active substances from natural plants has become a research hotspot.

*Lycium barbarum* has a great edible and medicinal value. As one of the most important bioactive ingredients in *Lycium barbarum*, *Lycium barbarum* polysaccharide (LBP) has multiple biological functions and pharmacological effects, such as anti-oxidation^4^, strengthening the barrier^5^, anti-tumor^6^, anti-inflammatory^7^, and regulating intestinal flora^8^. Recently, LBP was reported to effectively improve the clinical condition of RA by maintaining the bone integrity of type-II collagen-induced arthritis (CIA) rats and reducing the CIA-stimulated inflammatory mediators^9^. LBP can also affect the proliferation of chondrocytes and reduced arthritis^10^. Therefore, LBP is clearly a potential active compounds for RA treatment with low toxicity and low side effects. However, due to the complicated pathogenesis of RA, the mechanism of LBP on RA relief remains unclear.

LBP belongs to the non-starch polysaccharides (NSPs), which cannot be directly digested and absorbed by the body. NSPs will directly enter the large intestine, where they will be broken down by intestinal flora. More and more evidences prove that RA is closely related intestinal microecology^11^. Literatures showed enteric malnutrition and intestinal flora imbalance were typical features of RA^12^, while restoring intestinal homeostasis was proposed to be a therapeutic strategy to reduce RA^13^.

Intestinal microflora participated in the epigenetic shaping of host genes^14^, and modulated gene expression in intestinal epithelium^15^. But it is still elusive whether LBP modulated the gut microbiota, and how these gut bacteria played a role in RA alleviation. The aim of this study is to explore the potential mechanism of RA alleviation by LBP from the aspects of intestinal flora reshaping and intestinal epithelium gene regulation.

## Results

### RA-caused paw swell and joint pathological change were relieved by LBP

**Fig. 1:**
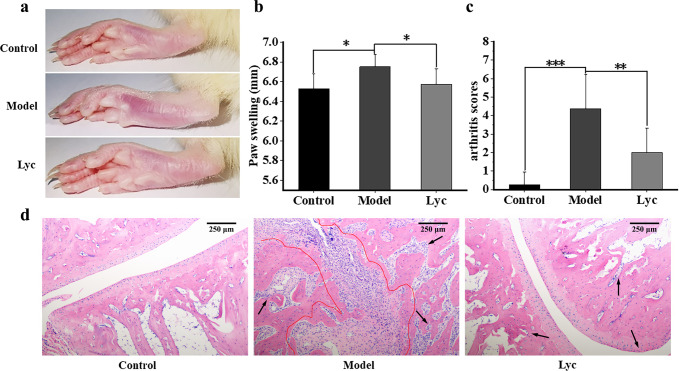
The severity of rheumatoid arthritis. **a** Joint swell of the left hind foot. **b** The diameter of paw swell in each group (mm). **c** The arthritis score of rats in each group. The error bars on the bar charts represented the standard deviations. The “*” indicated the *P* value was less than 0.05, “**” indicated the *P* value was less than 0.01, and “***” indicated the *P* value was less than 0.001. **d** Pathological sections of joint after HE staining in each group. Black arrow: diffusion or infiltration of inflammatory cells. Red circle: synovial hyperplasia and hypertrophy and formation of pannus. The magnification was 100×. The length of the scale bar was 250 μm.

Paw swell and arthritis score were used to assess the severity of arthritis. Paws of rats in the Model group were swollen during the whole experiment (Fig. 1a) as indicated by the significant larger diameter of paws (Fig. 1b) and higher arthritis score (Fig. 1c) comparing with those in the Control group (*P* < 0.01). Joint structure of rats in the Model group was abnormal, along with the infiltration of inflammatory cell, synovial and fibrous tissue hyperplasia, and the occurrence of synovial hypertrophy and pannus (Fig. 1d).

The administration of LBP relieved the severe arthritis (Fig. 1a). Comparing with the Model group, the average diameter of paw in the Lyc group significantly reduced (Fig. 1b) implying the paw swell was greatly improved. Similar result was also obtained from the arthritis score (Fig. 1c). In addition, rats in the Lyc group had less inflammatory infiltration, and no large areas of synovial hyperplasia, and did not form pannus as shown in hematoxylin and eosin (HE) staining (Fig. 1d).

### LBP reduced the content of pro-inflammatory and recovered the content of anti-inflammatory cytokines

**Fig. 2:**
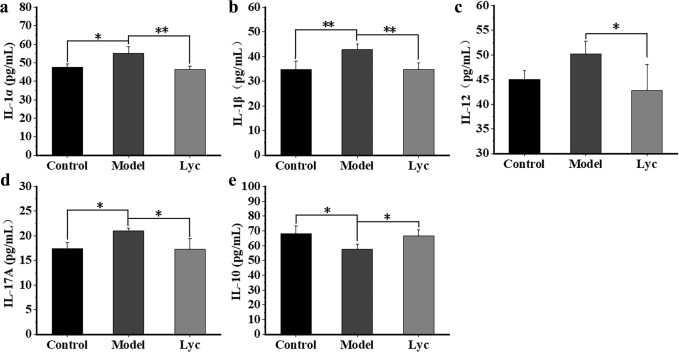
The contents of cytokines in the serum. **a** IL-1α, **b** IL-1β, **c** IL-12, **d** IL-17, and **e** IL-10. The error bars on the bar charts represented the standard deviations. The “*” indicated the *P* value was less than 0.05 while “**” indicated the *P* value was less than 0.01.

Pro-inflammatory factors including IL-1α, IL-1β, IL-12 and IL-17 abnormally increased while anti-inflammatory factor abnormally decreased in RA. Among them, both IL-1 and IL-17 are considered as important indicators in the pathogenesis of RA. They can aggravate autoimmune pro-inflammatory responses, promote synovial hyperplasia, and lead to cartilage erosion^16^. In our experiments, the contents of pro-inflammatory cytokines IL-1α, IL-1β, IL-12 and IL-17 in the Model group were significantly higher than those in the Control group (Fig. 2a–d), while the anti-inflammatory cytokine IL-10 was on the contrary (Fig. 2e). After the administration of LBP, the contents of serum IL-1α, IL-1β, IL-12 and IL-17 in the Lyc group significantly reduced (Fig. 2a–d), while the content of IL-10 was recovered and tend to become normal (*P* < 0.05) (Fig. 2e). Together with the results of the pathological changes, LBP could significantly alleviated RA.

### LBP altered specific intestinal bacteria that correlated to the inflammatory cytokine level and RA symptoms

**Fig. 3:**
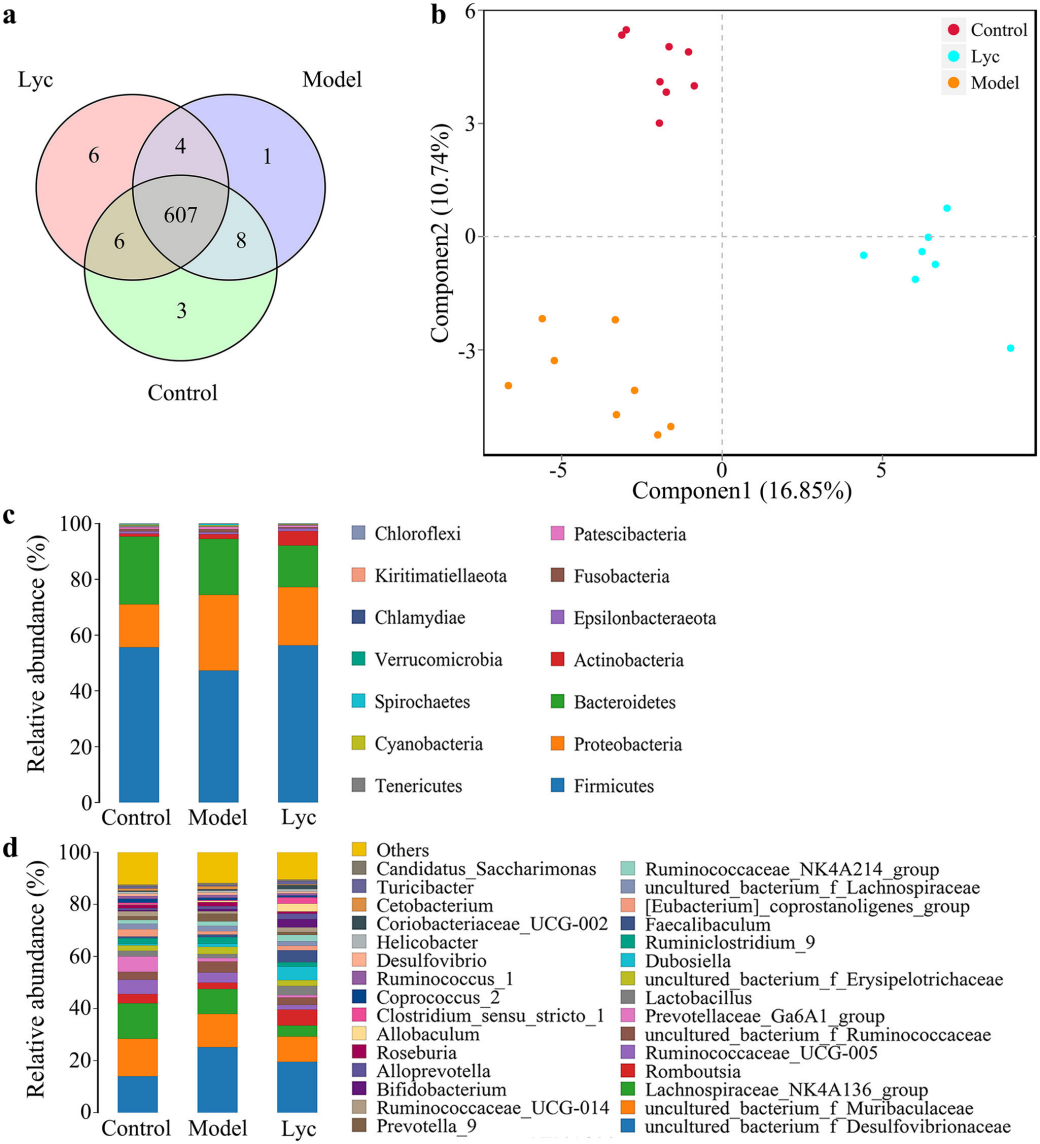
Metagenomics information of intestinal microflora among the Control, Model and Lyc group. **a** The Venn diagram on the OTU level among groups. **b** PLS-DA plot among groups. **c** The relative abundance of microflora on the phylum level and **d** on the genus level (*n* = 8 for Control and Model group, *n* = 7 for Lyc group).

On the whole, a total of 1,840,370 valid reads were obtained from all samples. The intestinal microflora on the Operational Taxonomic Unit (OTU) level showed that all groups shared the vast majority of OTU, but it’s worth noting that 6 unique OTUs in the Lyc group (Fig. 3a). Moreover, the intervention of LBP reconstructed intestinal microflora as indicated by the apparent distance among different groups (Fig. 3b). On the phylum level (Fig. 3c), the relative abundance of *Fusobacteria* was higher in the Model group than that in the Lyc group, which means the LBP intervention significantly altered the growth of *Fusobacteria* (Table 1). As for the genus level, when compared with the Model group, the Lyc group showed a lower relative abundance of *Lachnospiraceae*_NK4A136_group and uncultured_bacterium_ f_*Ruminococcaceae*, and a higher relative abundance of specific bacteria, including *Romboutsia*, *Lactobacillus*, *Dubosiella* and *Faecalibaculum* (Fig. 3d). The altering of microbial compositions manifested the LBP significantly reconstructed the intestinal microflora in RA rats.

**Table 1:** The bacteria with a significant difference of relative abundance on the genus level between the Model group and the Lyc group (*p* < 0.05).

Bacteria name	*P* value	Bacteria abundance^1^
*Fusobacteria*	0.0002	DOWN
*Lachnospiraceae*_*NK4A136*_group	0.0073	DOWN
uncultured_bacterium_f_*Ruminococcaceae*	0.0237	DOWN
*Faecalibaculum*	0.0155	UP
*Romboutsia*	0.0237	UP
*Lactobacillus*	0.0101	Up
*Dubosiella*	0.0029	Up

Noted: The superscript “1” indicated the relative abundance was down-regulated or up-regulated in the Lyc group when compared with the Model group.

**Fig. 4:**
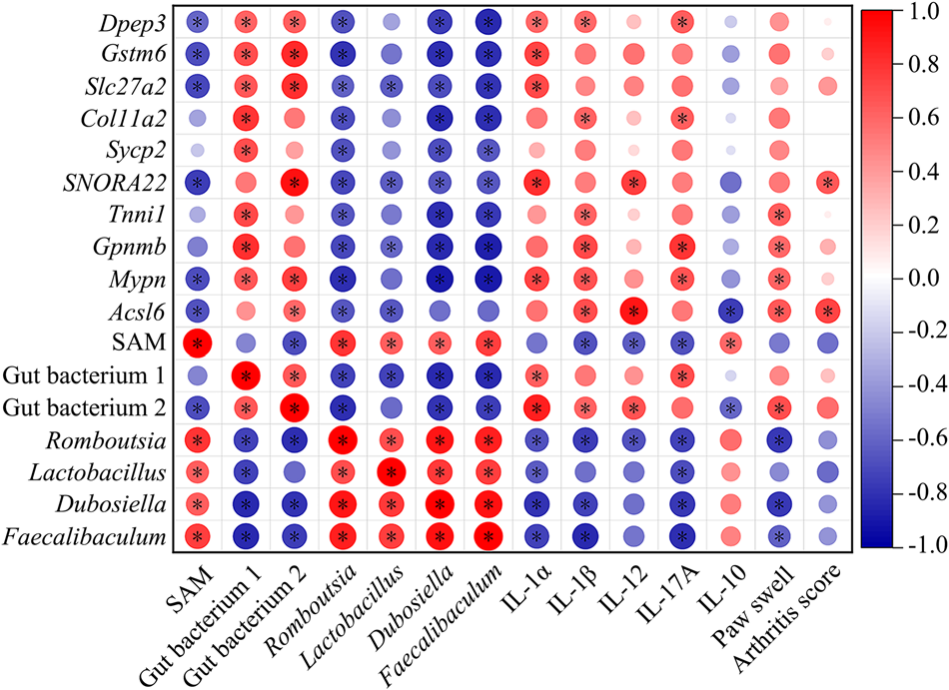
Correlation heatmap among the joint status, the contents of SAM and cytokines, the abundances of specific gut bacteria and the FPKM value of differentially expressed genes. Noted that, Gut bacterium 1 represents the uncultured_bacterium_f_*Rumino-coccaceae*; Gut bacterium 2 represents the *Lachnospiraceae*_*NK4A136*_group.

Correlation analysis revealed that both *Lachnospiraceae*_NK4A136_group and uncultured_bacterium_f_*Ruminococcaceae* were positively correlated to the content of pro-inflammatory cytokines, paw swelling and arthritis scores while negatively correlated to the content of IL-10. In contrast, *Romboutsia*, *Lactobacillus*, *Dubosiella* and *Faecalibaculum* were negatively correlated to those factors except the IL-10 (Fig. 4). These results indicated elevating abundance of *Romboutsia*, *Lactobacillus*, *Dubosiella* and *Faecalibaculum* by LBP intervention played a role in the inflammation reduction and the RA alleviation.

### LBP altered the transcriptome of colonic epithelial tissue

**Fig. 5:**
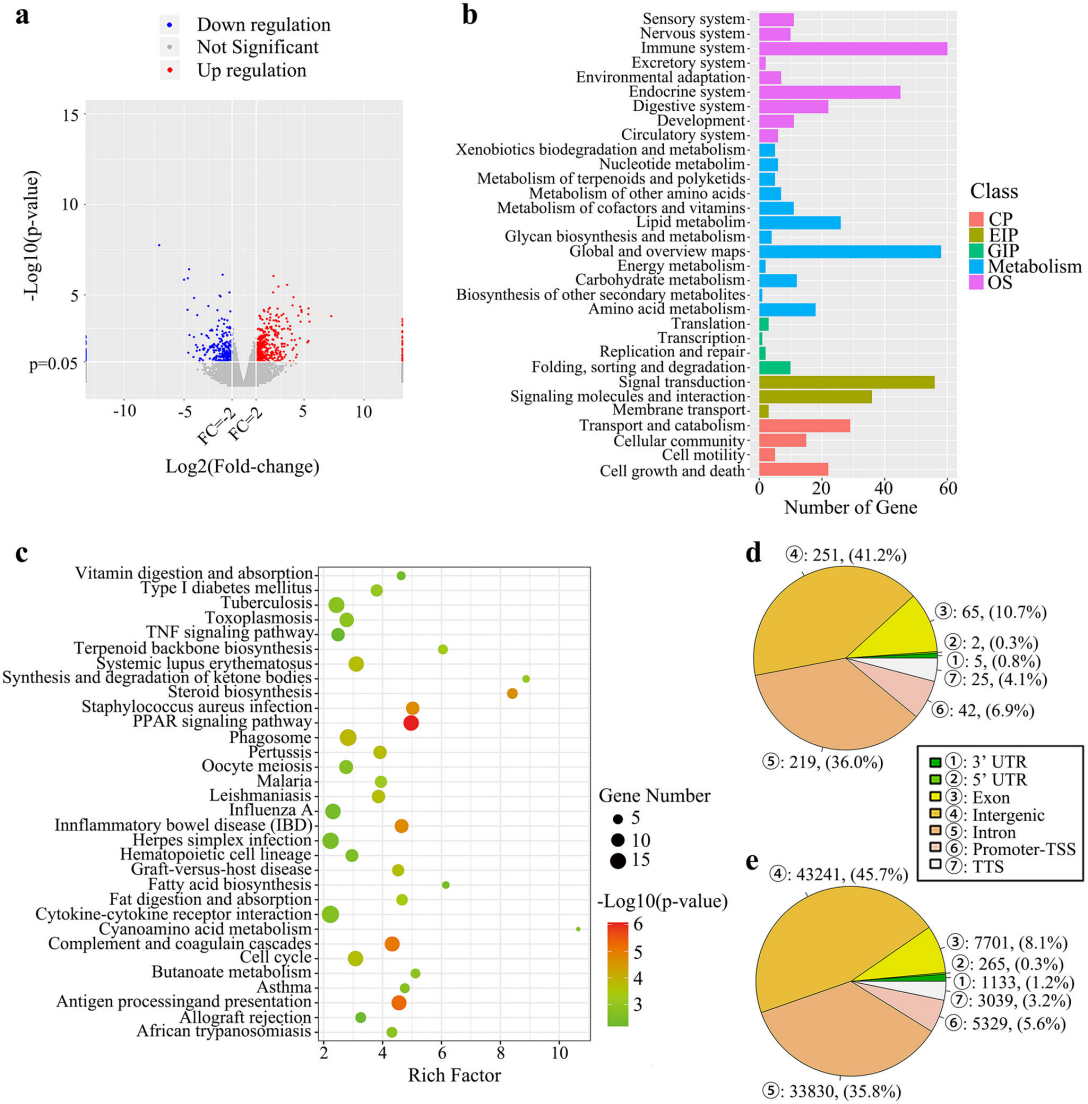
Transcriptomics and methylomics information between the Model and Lyc group. **a** Volcano plot of differentially expressed genes (DEGs) between groups. **b** The top 30 KEGG classes which DEGs were classified. Noted that, CP: Cellular Processes, EIP: Environmental Information Processing, GIP: Genetic Information Processing, OS: Organismal Systems. **c** The KEGG pathways which DEGs significantly enriched. **d** Distribution of differentially methylated regions (DMR) and **e** differentially methylation sites (DMS) on different gene elements. Noted that, different colors and numbers in circle represented different gene elements.

In total, 462 genes were differentially up-regulated and 293 were down-regulated in the Lyc group when compared with those in the Model group (Fig. 5a). These differentially expressed genes (DEGs) can be classified into multiple KEGG classes (Fig. 5b, top 30 classifications in level 2 were shown). Among all classifications, the immune system covered the largest number of DEGs, followed by global and overview maps and signal transduction. Further evaluation for the enrichment analysis showed 32 KEGG pathways were significantly enriched (Fig. 5c). Nearly half of the pathways (15/32) belonged to the classification of ‘Human Diseases’, and 14/15 of these pathways related to infectious or immune disease. In addition, some inflammation-related pathways such as TNF signaling pathway, PPAR signaling pathway and cytokine-cytokine receptor interaction were also included. The transcriptomic results implied the intervention of LBP may have some impacts on immune-related genes in colonic epithelial tissue.

### LBP reprogramed DNA methylome, and thereby regulated the expression of specific genes to alleviate RA

According to the results of methylome, 697 differentially methylated regions (DMRs) annotating 375 genes were found between the Lyc and Model groups. Among them, 6.9% located in the promoter region (Fig. 5d). Additionally, 51,298 nucleotide sites were methylation-specific modified, and most of them are located in intergenic and intron, while 5.6% and 8.1% are located in promoter and exon regions, respectively (Fig. 5e). Hypermethylation, especially for that occurred in the promoter region, is thought to inhibit gene expression by preventing the transcription factors from combining with the gene^17,18^. Together with the results of transcriptome, 89 genes in the Lyc group were found to be in high methylation level and simultaneously showed suppressed expression when using the Model group as the control. We further screened out 10 genes including *Dpep3*, *Gstm6*, *Slc27a2*, *Col11a2*, *Sycp2*, *SNORA22*, *Tnni1*, *Gpnmb*, *Mypn* and *Acsl6* based on the number of methylation site and/or the region of methylation (Table 2). All these genes were found to have a higher fragments per kilobase of exon per million mapped reads (FPKM) value but a lower methylation level in the Model group while they have a lower FPKM value but a higher methylation level in the Lyc group.

**Table 2:** Differential genes (*P* < 0.05 in both transcriptome and methylome) with hypermethylation and suppressed expression after the intervention of LBP.

Gene name	Log_2_FC^1^	FC^2^	Total methylated sites	Region	
				Promoter	Intron (TTS)
*Dpep3*	−3.4227	2.0922	2	2	0
		1.3248			
*Gstm6*	−1.0884	2.4611	2	2	0
		2.0462			
*Slc27a2*	−4.1620	14.3865	2	2	0
		25.9880			
*Col11a2*	−1.1298	6.3871	3	3	0
		1.5333			
*Sycp2*	−1.6692	1.6122	1	1	0
*Mypn*	−2.6812	1.4219	1	1	0
*Tnni1*	−1.7056	1.6398	1	1	0
*Gpnmb*	−1.4783	1.7950	1	1	0
*SNORA22*	−2.3002	1.2696	1	0	1
*Acsl6*	−3.5799	1.2940	1	0	1

FC is an abbreviation of fold change; TSS is an abbreviation of transcription termination sites; the FC with superscript “1” is calculated by dividing the average FPKM value of a gene in the Lyc group by that in the Model group; the FC with superscript “2” is calculated by dividing the average methylated degree of specific location in a gene in the Lyc group by that in the Model group.

The expression of these genes was positively correlated to either the content of certain pro-inflammatory cytokines or the paw swell and arthritis score with statistically significance (Fig. 4). Specifically, the correlation between the gene expression of *Dpep3*, *Mypn* and the content of IL-1α, IL-1β, IL-17A; *Col11a2*, *Gpnmb* and IL-1β, IL-17A; *SNORA22* and IL-1α, IL-12; *Acsl6* and IL-1β, IL-12; *Gstm6* and IL-1α; *Slc27a2* and IL-1α; *Tnni1* and IL-1β was significantly positive (*P* < 0.05). Additionally, the expression of *Acsl6* was negatively correlated to the content of IL-10 with significance (*P* < 0.05). Furthermore, the expression of *Tnnil1*, *Gpnmb*, *Mypn* and *Acsl6* was positively correlated to the paw diameter (paw swell) while the expression of *SNORA22* and *Acsl6* was positively correlated to the arthritis score (*P* < 0.05). Therefore, the downregulation of these genes was closely connected to the inflammation and RA alleviation.

### The methylome reprograming was modulated through direct methyl donor SAM potentially generated by LBP-intervened gut microbiota

Previous studies have shown that intestinal microorganisms affect the expression of host genes by impacting the epigenetic modification of host genes^19,20^. Therefore, we speculate that the suppressed expression of those specific genes mentioned above due to the DNA hypermethylation could be related to the gut microbiota intervened by LBP. Based on the results of the correlation analysis, the mRNA content of *Acsl6* was negatively correlated to the relative abundances of *Romboutsia* and *Lactobacillus* while the mRNA contents of both *Slc27a2* and *SNORA22* were negatively correlated to the abundances of all four bacteria (*Romboutsia*, *Lactobacillus*, *Dubosiella* and *Faecalibaculum*) with significance. The mRNA contents of other genes were also negatively correlated to the abundances of *Romboutsia*, *Dubosiella* and *Faecalibaculum* (*P* < 0.05) (Fig. 4).

**Fig. 6:**
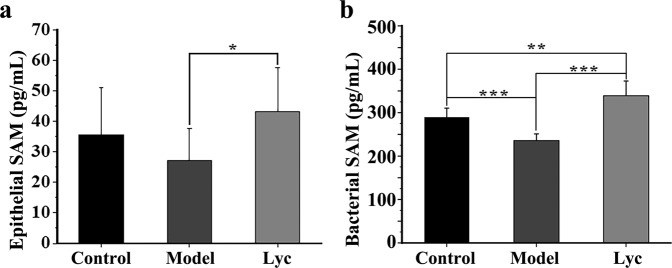
The contents of S-adenosyl methionine (SAM) of each group. **a** SAM contents in colonic epithelial tissue and **b** intestinal contents. The error bars on the bar charts represented the standard deviations. The “*” indicated the *P* value was less than 0.05, “**” indicated the *P* value was less than 0.01, and “***” indicated the *P* value was less than 0.001.

Further identification of the substance accounted for hypermethylation was applied. The results showed that the content of SAM in the colonic epithelium in the Lyc group significantly increased compared with that in the Model group (Fig. 6a) (*P* < 0.05). Judging from the correlation analysis, we found a strong positive correlation between the content of epithelial SAM and the relative abundance of 4 specific gut bacteria (*Romboutsia*, *Lactobacillus*, *Dubosiella* and *Faecalibaculum*) (Fig. 4). In addition, the content of SAM inside the intestinal contents (bacterial SAM) significantly was higher in the Lyc group than the Model group (Fig. 6b) (*P* < 0.05). These results may show a possibility that the additional production of SAM (the direct methyl donor possibly caused hypermethylation in specific genes) in the Lyc group was due to the increase of those 4 bacteria.

## Discussion

RA is a chronic disease ubiquitous all over the world. It is the result of complex interactions among genes, environmental and hormonal factors and the immune system^21^. In our experiment, LBP relieved the typical features of RA in rats, including the reduction of paw swell, arthritis scores and the contents of pro-inflammatory cytokines such as IL-1α, IL-1β, IL-12 and IL-17, and the recovery of the content of anti-inflammatory cytokine IL-10. In addition, LBP improved the pathological manifestations of joint and cartilage in RA rats. Multi-omics analysis also manifested that LBP reshaped the gut microbial structure, specifically the modulation of the relative abundance of *Romboutsia*, *Lactobacillus*, *Dubosiella*, *Faecalibaculum*, *Lachnospiraceae*_NK4A136_group and uncultured_bacterium_f_*Ruminococcaceae*, and regulated the gene expression in colonic epithelium, especially for the suppressed expression of *Dpep3*, *Gstm6*, *Slc27a2*, *Col11a2*, *Sycp2*, *SNORA22*, *Tnni1*, *Gpnmb*, *Mypn* and *Acsl6* due to the DNA hypermethylation.

It is widely accepted that intestinal microflora is a factor affecting metabolic homeostasis and the immune system^22^. The researcher has proposed the concept of the gut-joint axis, implying connections between intestinal microflora and RA. For instance, in the early stages of RA, the number of *Bifidobacteria* and *Bacteroidetes* decreased while the number of *Prevotella* increased^23^. It is also reported that the gut microbiome in the new-onset RA was characterized by an increase of *P. Copri* and a reduction of *Bifidobacteria*^24^. Therefore, the alleviation of RA by LBP should be attributed to its capability for gut microbiota modulation. In this current study, we found specific bacteria including *Lachnospiraceae*_NK4A136_group, uncultured_ bacterium_f_*Rumino-coccaceae*, *Romboutsia*, *Lactobacillus*, *Dubosiella* and *Faecalibaculum* were regulated. Among these bacteria, *Fusobacteria* and *Lachnospiraceae* are reported to associate with inflammatory bowel disease^25–27^, while inflammatory bowel disease is considered as the predisposing factor of RA^28^. It seems that LBP could alleviate RA by reducing some susceptibility factors caused by gut microbiota. Additionally, LBP recovered the abundance of *Romboutsia* and *Faecalibacterium*, whose loss could lead to intestinal inflammation and incompleteness of intestinal epithelium. In this regard, LBP might facilitate the colonization of beneficial microorganisms in the intestine, enhance the intestinal barrier function, relieve the intestinal permeability, maintain intestinal integrity and intestinal health, and promote intestinal development^29^. LBP intervention also elevated the abundance of *Lactobacillus*, which is a characteristic of bacteria in rheumatoid joint diseases. It is reported that joint swelling and bone destruction can be improved by increasing the abundance of *Lactobacillus*^30^. Particularly, *L*. *salivarius* or *L*. *plantarum* showed RA amelioration from clinical manifestations^31^, and *L*. *fermentum* as well as *L*. *swissericus* also alleviated RA^32^, and even showed a certain ability for RA prevention^33^. Hence, the LBP-intervened microbiota should play an important role in the mechanism of RA alleviation.

The alteration of microbial structure caused by NSPs’ consumption was commonly accompanied with the change of gene expression in the intestinal epithelium. As predicted, the expression of over 700 genes were differentially regulated. We further found over 60% of these DEGs were methylated modified. Methylation modification is one of the epigenetic changes which altered the DNA configuration but not the nucleotide sequence to affect the expression of genetic material^34^. Gene expression is negatively correlated with the degree of methylation, and genes in hypermethylation are transcriptionally suppressed^35^. Since LBP lessened the RA, we focused on those down-regulated genes caused by DNA hypermethylation and eventually screened out 10 genes including *Dpep3*, *Gstm6*, *Slc27a2*, *Col11a2*, *Sycp2*, *SNORA22*, *Tnni1*, *Gpnmb*, *Mypn* and *Acsl6*, which can be divided into 3 categories.

Genes related to the alleviation of RA and RA-associated lesions including *Col11a2*, *Acsl6*, *Gpnmb* and *SNORA22*, can be classified into category A. Among them, *Col11a2* is a gene encoding type XI collagen, which plays a key role in maintaining the characteristics of the cartilage matrix^36^. Studies have shown that the expression of *Col11a2* was significantly increased in the cartilage of patients with arthritis, the overexpression of *Col11a2* promoted the destruction of articular cartilage and chondrocyte necrosis^37^. LBP reduced the expression of *Col11a2*, which slowed down the degradation of articular cartilage and relieved RA. In addition, the overexpression of *Acsl6* in chondrocytes induced the typical characteristics of arthritis, leading to the down-regulation of type-II collagen and the destruction of articular cartilage^38^. Since LBP had caused the high methylation and low expression of *Acsl6* gene, it should inhibit the destruction of articular cartilage, and thus alleviated arthritis. Angiogenesis was reported to positively correlate with the severity of RA^39^. As a gene highly expressed in tumors^40^, *Gpnmb* promoted angiogenesis^41,42^. Thus, its suppressed expression after LBP consumption may reduce the angiogenesis and further alleviate RA. The overexpression of *SNORA22* positively correlated with arthritis because it contributes to cell invasion and tumor metastasis, while cell erosion can aggravate the disease manifestations of RA^43^. In our experiment, LBP inhibited *SNORA22* gene expression by elevating the methylation level, and may eventually improve RA.

Genes related to the inflammation including *Slc27a2*, *Gpnmb*, *Gstm6* and *Acsl6*, can be classified into category B. Obesity can be considered as a chronic inflammation with low grade, and *Slc27a2* was reported to be a potential marker of obesity^44^. Its expression was positively correlated to both content of IL-6 and TNF-α in peripheral blood mononuclear cells isolated from obese human^45^. *Gpnmb* was highly expressed in macrophage, and its content was increased in pro-inflammatory conditions^46^. It promoted the production of TNF-α in in vitro cell test^47^. The LBP intervention in the current research lowered the expression of both *Slc27a2* and *Gpnmb* by remodeling their DNA methylome, and subsequently relieved the inflammation. *Gstm6* and *Acsl6*, were also regarded as the candidate genes related to inflammation in hepatic lesions such as hepatitis and liver cancer^48,49^. However, interestingly, the low expression of these 2 genes enhanced the inflammation were reported, which was different from our results. This is probably due to different disease models applied in those reports. But further study will be needed to clarify the exact function of these 2 genes in RA.

Similarly, the rest of genes including *Dpep3*, *Sycp2*, *Tnni1* and *Mypn*, which can be classified into category C, should also be addressed since no literatures reported their connections to RA or inflammation. Nevertheless, based on our current study, we believed they also played an important role in RA alleviation.

Taken together, the suppressed expression of these 10 epithelial genes due to their hypermethylation by LBP intervention may lower the content of proteins that associated with RA-caused lesions or inflammatory cytokines, and their transportation towards joint through blood flows were then possibly reduced. Therefore, LBP intervention may alleviate RA through gut-joint axis.

DNA methylation modulated the gene expression. As the direct methyl donor, SAM is critical in the process of DNA methylation. It transfers its own active methyl groups to specific bases in the DNA strand to methylate DNA and directly affects the degree of DNA methylation. Our results demonstrated LBP consumption recovered the insufficient SAM caused by RA, ultimately leading to DNA hypermethylation and gene hypo-expression. It should be noted that, SAM is also a common and important metabolite of intestinal microflora. Studies have confirmed that SAM from gut bacterial fermentation could be available to the host, and it is a significant, yet underestimated source of SAM for the host^50^. As one type of the NSPs, LBP reconstructed the gut microbiota, especially for the elevation of *Lactobacillus*, and could be subsequently metabolized into SAM. This was supported by some documents reporting that SAM was produced by in vitro fermentation of *Lactobacillus*^51^. In addition, *Lactobacillus* was listed as excellent SAM-producing bacteria among multiple SAM-producing probiotics^50,51^. Therefore, the hypermethylation of specific genes in intestinal epithelium should be the result of the SAM content recovery. This recovery possibly owing to the alteration of gut microbiota, especially for the increased abundance of several gut bacteria such as *Lactobacillus* after LBP intervention.

Collectively, the intervention of LBP relieved RA effectively. The underlying mechanism may lie in the aspect that LBP can increase the SAM content by reshaping the intestinal flora, resulting in DNA hypermethylation and suppressed expression of certain genes related to RA, thus ameliorating RA. Specifically, the LBP intervention reduced the abundances of *Lachnospiraceae*_NK4A136_group and uncultured_ bacterium_f_*Rumino-coccaceae*, and increased the abundances of *Romboutsia*, *Lactobacillus*, *Dubosiella* and *Faecalibaculum*. The content of the direct methyl donor SAM possibly generated from the above elevating gut bacteria was raised, and subsequently increased the methylation degree of specific colonic epithelial genes reportedly associated with RA including *Dpep3*, *Gstm6*, *Slc27a2*, *Col11a2*, *Sycp2*, *SNORA22*, *Tnni1*, *Gpnmb*, *Mypn* and *Acsl6*, and further inhibited their expressions. The symptoms of RA including the paw swell, joint pathological change and inflammatory status were eventually improved possibly through the gut-joint axis. The current study lacked the direct confirmation on the capability of SAM production by those bacteria with increased relative abundance, as well as the elucidation about the undefined function during RA alleviation of several genes. Therefore, further relevant studies are still needed in the future.

## Methods

### Reagents and materials

LBP was purchased from Shanghai Yuanye Bio-Technology Co., Ltd. (Shanghai, China). Our previous report have shown that the monosaccharide composition ratio of LBP is Mannose: Ribose: Rhamnose: Glucuronic acid: Galacturonic acid: Glucose: Galactose: Xylose: Arabinose=6:1:2:1:9:38:10:12:21^46^. Incomplete Freund’s adjuvant (IFA) and bovine type II collagen were purchased from Beijing Chondrex International Trade Co., Ltd. (Beijing, China).

### Animal housing

All procedures were approved by the Institutional Animal Care Committee of Jinan University (2019827-02), and in accordance with the approved guidelines. Twenty-four specific-pathogen-free (SPF) female Wistar rats (8 weeks old) were purchased from Laboratory Animal Center of Southern Medical University (Guangzhou, Guangdong, China). Rats were raised in controlled standard barrier environment with temperature control (23 ± 2 °C), humidity control (55 ± 5%) and a 12-h light/12-h dark cycle at the Animal Center of Jinan University. During the experiment, rats were allowed to eat (AIN-93M) and drink (sterile distilled water) freely.

### Induction of the type-II collagen arthritis models

According to the instructions of manufacturer, an equal volume of bovine type II collagen was mixed with IFA. The above mixture was totally emulsified by shaking before used. Rats were immunized with two subcutaneous injections with an interval of 7 days. For the first time, 0.2 mL of emulsion was injected at 1.5 cm away from the caudal end of the rats; and for the second time, 1.5 mL of the emulsion was injected subcutaneously at 3 cm from the caudal end of the rats.

### Grouping, treatment and sample preparation

**Fig. 7:**
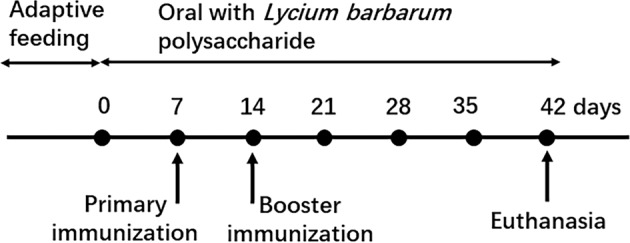
The time flow of modeling, gavage, and euthanasia during animal experiments. After adaptive feeding, bovine type II collagen and IFA mixture were subcutaneous injected to rats at day 7 for primary immunization, and at day 14 for booster immunization. The experiment lasted for 42 days, and the LBP solution and sterilized distilled water were orally administered to rats during this period.

After 7 days of adaption, rats were randomly divided into three groups (*n* = 8 for each), which were the Control, Model and Lyc groups. For rats in the Lyc group, LBP solution (400 mg·kg^−1^) was given once a day by gavage, for those in the Control and Model groups, equal volume of sterilized distilled water was used to replace the LBP solution, and the administration of LBP lasted for 42 days (Fig. 7)^52^.

The dose of 400 mg·kg^−1^ body weight for rats is equivalent to the dose of 63.49 mg·kg^−1^ body weight for human. On day 7 and 14, the rats were immunized by bovine type II collagen and IFA mixture according to the methods described above to establish RA models (the rats in the Control group were injected with the equivalent saline). On day 42, the rats were fasted overnight, and anesthetized with pentobarbital sodium on day 43 by intraperitoneal injection (50·mg kg^−1^ body weight) (Qiyun, Guangzhou, Guangdong, China). The blood samples were collected from the abdominal aorta and then the serum samples were obtained by centrifuged at 12,000 rpm for 30 min. After the rats were sacrificed, the ceca were removed and the cecal content of each sample was transferred into a sterile EP tube. Joint soft tissues were also removed and then packed with the saline-rinsed gauze. Colonic epithelial tissues were scraped from the inner side of colon, placed into the sterile folded silver paper and then stored in the liquid nitrogen immediately. All samples except the colonic epithelial tissues were stored in a cryogenic refrigerator at −80 °C.

### Assessment of CIA

The diameter of rat’s hind paws, which is one of the indices to evaluate the severity of CIA model, were measured with a vernier caliper every 7 days. From the 7^th^ day, the arthritis score which graded from 0 to 4 were recorded with the same frequency to the measurement of hind paw diameters^53^. The scoring criteria was listed at Table 3.

**Table 3:** The scoring criteria of arthritis score.

Severity score	Degree of inflammation
0	No redness and swell
1	Redness and swell of the knuckles
2	Redness and swell of the knuckles and plantars
3	Redness and swell below the ankles
4	All the ankles, plantars and knuckles

The final score is the sum of four feet, which means the possible maximum score for each rat is 16.

### HE staining and pathological evaluation

After euthanasia, the rats’ hind feet were collected. The joint tissues were removed from the hind feet, fixed in 4% (v/v) neutral paraformaldehyde and then decalcified in 10% EDTA for 30 days. They were then embedded in paraffin. Joint tissues were sectioned at 5 μm of thickness, stained with HE. The HE-stained sections were evaluated in terms of inflammatory cell infiltration, synovial hyperplasia, fibrous tissue hyperplasia and pannus formation.

### Measurement of cytokine contents in serum

The serum was stored at −80 °C until analysis. The contents of IL-1α, IL-1β, IL-10, IL-12 and IL-17 in serum were measured with ELISA kits according to the manufacturer’s instructions (Nanjing SenBeiJia Biological Technology Co., Ltd, Nanjing, China and Jiangsu Enzyme Industry Co., Ltd., China).

### 16S rDNA sequencing and bioinformatics analysis

Based on the previous research, the cecal bacterial DNA of each rat was extracted and analyzed^54^. Briefly, V3-V4 regions of the bacterial 16S rRNA gene were amplified by polymerase chain reaction (PCR), then PCR amplification products were purified, quantified and homogenized to construct sequencing libraries. The Illumina HiSeq Sequencing Platform (Illumina HiSeq 2500) was applied to sequence the library that pass the quality inspection. Then the library was converted into the original sequenced sequence (Sequenced Reads) by Base Calling analysis, splicing and filtering Reads, clustering OTUs, and performing species annotation and abundance analysis, alpha diversity analysis (Alpha Diversity), beta diversity analysis (Beta Diversity) and significant species difference analysis.

### Analysis of transcriptome in colonic epithelium

Total RNA was extracted with RNeasy Mini Kit (QIAGEN, Shanghai, China), and RNA integrity (RIN) was evaluated with an Agilent Bioanalyzer 2100 (Agilent technologies, Santa Clara, CA, USA). The qualified total RNA was purified with RNAClean XP Kit (Beckman Coulter, Shanghai, China) and RNase-Free DNase Set (QIAGEN, Shanghai, China). The mRNA was isolated from purified total RNA and the genomic mRNA was fragmented. The cDNA was synthesized with random hexamers and then subjected to end repair. The libraries were size-selected for cDNA target fragments of 200–300 bp with 2% low-range ultra-agarose gels followed by PCR amplification using Phusion DNA polymerase for 15 PCR cycles. After quantification using a Qubit® 2.0 Fluorometer and Agilent Bioanalyzer 2100, the paired-end RNA-seq sequencing library was sequenced with the IlluminaHiSeq 4000.

To identify the DEGs between two different groups, the expression level of each transcript was calculated according to the FRKM method. DEGs were selected with the following criteria: *P* value should be less than 0.05 and the absolute value of base-2 logarithm to fold change should be larger than 2. The R statistical package software Empirical Analysis of Digital Gene Expression in R (EdgeR) was utilized for the differential expression analysis.

### Analysis of methylome in colonic epithelial cells

DNA from colonic epithelial cell samples was extracted with a gDNA Extraction Kit (Omega, Guangzhou, Guangdong, China). Then, the recommended Methylation-Sequencing protocol and specific enzymes were used to repair the ends, adenylate the 3’ ends and ligate methylated adaptors. The samples were then hybridized to biotinylated RNA baits and purified with streptavidin beads (New England BioLabs, Beijing, China). The remaining target sequences were bisulfite converted with an EZ DNA Methylation-Gold kit (Zymo Research, Irvine, CA, USA) as described in the Methylation-Sequencing protocol. The sequencing library was established by PCR amplification. Finally, the libraries were indexed and randomly pooled into multiplexes of four samples, and then sequenced using one pool per lane on the Illumina Hiseq X ten Platform (150 bp paired-end reads, Illumina Technologies, Shanghai, China). Sequencing was completed at the Shanghai Biotechnology Corporation (Shanghai, China).

The quality of raw data was evaluated with FastQC v0.11.5 (http://www.bioinformatics.babraham.ac.uk/projects/fastqc/). The sequencing quality (Q) was calculated with the formula of Q = −10Log10E, where E stands for the sequencing error rate. The clean reads were obtained after the filtration using Trim_galore v0.4.1 (http://www.bioinformatics.babraham.ac.uk/projects/trim_galore/) with the following procedures: 1) remove the reads with low quality (Q < 20 in 3’ end or Q > 20 but accounted for less than 50%); 2) remove all adaptor sequences inside the reads; 3) remove reads with length less than 70 and unpaired. The clean reads of genome were compared by Bismark v0.15.0 using the genome of Rattus norvegicus as reference. ^55^. The methylation sites were scanned by Bison v0.4.0 with default setting^56^. Differential methylation sites (DMS) and DMR were detected and annotated by the tool of dispersion Shrinkage for Sequencing data (DSS) in the Bioconductor package^57^. Those sites with *P* < 0.05 and difference of methylation degree>10% were considered as DMS, and the region within which all CpG sites were with *P* < 0.05 was considered as DMR. The DMS were mapped into Gene Ontology (GO) database, and screened by hypergeometric test. Significant enrichment can be considered when *P* < 0.05.

### Detection of S-adenosyl methionine (SAM) content in colonic epithelial tissue and intestinal contents

The mixture of 1 g colonic epithelial tissue and 200 μL saline or 0.1 g intestinal contents and 900 μL saline was fully homogenized with a high-throughput tissue homogenizer, and centrifuged at 3000 rpm for 15 min, and the supernatant was then carefully collected. The content of SAM was measured by corresponding ELISA kit according to the manufacturer’s instructions (Jiangsu Enzyme Industry Co., Ltd., China). The epithelial samples and the standards were added to the enzyme-labeled coating plate. The plate was incubated at 37 °C for 30 min, and then repeatedly washed with the washing solution for 5 times. After that, 50 μL of HRP-conjugate reagent was added to the plate. The plate was incubated at 37 °C for 30 min and washed for 5 more times. After that, 50 μL of chromogenic solution A and chromogenic solution B were added to the plate, and then the plate was kept in dark for ten minutes at 37 °C. Finally, the stop solution was added into the plate, and the absorbance was detected at the wavelength of 450 nm with a grating-type multifunctional microplate detector.

### Statistical analysis

The statistical analysis of the bioinformatics data was performed using R software v3.3.1 with the default setting. Other analysis was performed with SPSS 20.0 software. The results were presented as the mean values with standard deviations. The normal distribution of data was checked by Shapiro-Wilk test when the significance was larger than 0.05. One-way analysis of variance (ANOVA) with Tukey’s test was applied to analyze the significance among different groups. Correlation heatmap was generated using the software of Origin 2021. Statistical significance was set at a *P* value less than 0.05.

## Data Availability

The datasets presented in this study can be found in online repositories. The names of the repository/repositories and accession number(s) can be found below: https://www.ncbi.nlm.nih.gov/, PRJNA844171 and PRJNA846997. The other data from the current study are available from the corresponding authors on reasonable request.
